# Survival in patients with HR+/HER2− metastatic breast cancer treated with initial endocrine therapy versus initial chemotherapy. A French population-based study

**DOI:** 10.1038/s41416-020-0979-3

**Published:** 2020-07-17

**Authors:** Julien Simon, Marie Chaix, Oumar Billa, Ariane Mamguem Kamga, Patrick Roignot, Sylvain Ladoire, Charles Coutant, Patrick Arveux, Catherine Quantin, Tienhan Sandrine Dabakuyo-Yonli

**Affiliations:** 1 Breast and Gynecologic Cancer Registry of Côte d’Or, Georges-François Leclerc Comprehensive Cancer Care Centre, 1 rue Professeur Marion, Dijon, France; 2 Biostatistics, Biomathematics, Pharmacoepidemiology and Infectious Diseases, Inserm U1181, Villejuif, France; 3 Medical Oncology Department, Georges-François Leclerc Comprehensive Cancer Care Centre, 1 rue Professeur Marion, Dijon, France; 4 Pathology Centre, 33 rue Nicolas Bornier, Dijon, France; 5 Surgical Oncology Department, Georges-François Leclerc Comprehensive Cancer Care Centre, 1 rue Professeur Marion, Dijon, France; 6 Center for Research in Epidemiology and Population Health, Inserm U1018, Villejuif, France; 7 CHRU Dijon, Service de Biostatistiques et d’Information Médicale (DIM), Dijon, 21000 France; 8 INSERM, CIC 1432, Univ Bourgogne Franche Comte, Dijon, France; 9 Inserm U1231 Lipids, Nutrition, Cancer Research Center, Dijon, France

**Keywords:** Breast cancer, Chemotherapy

## Abstract

**Background:**

According to international guidelines, endocrine therapy (ET) is the preferred option for hormone receptor-positive (HR+) HER2-negative (HER2−) metastatic breast cancer. In spite of clear recommendations, these are not strictly followed in daily practice. The objectives of this study were to investigate the effect of the first anti-metastatic treatment therapy choice on progression-free survival (PFS) and overall survival (OS).

**Methods:**

In this population-based study, we included patients with HR+/HER2− metastatic breast cancer recorded in the Côte d’Or Breast Cancer Registry. Differences in PFS and OS between patients initially treated with chemotherapy (CT) or ET were analysed in Cox proportional hazards models. In a sensitivity analysis, we used a propensity score (PS) to limit the indication bias.

**Results:**

Altogether, 557 cases were included, 280 received initial ET and 277 received initial CT. PFS and OS in patients initially treated with ET was improved significantly when compared to patients with initial CT (respectively, HR = 0.83 (95% CI 0.69–0.99) and HR = 0.71 (95% CI 0.58–0.86)). The results of the sensitivity analysis supported these findings.

**Conclusion:**

This study shows that treating patients with HR+/HER2− metastatic breast cancer with initial ET could provide a survival advantage in comparison with initial CT.

## Background

Breast cancer is the most common malignancy among women, representing almost a quarter of all cancer cases in females in Europe and worldwide^1^ and causing >12 thousand deaths in France in 2018.^2^

Thanks to detection at an earlier stage and the development of better treatments over the past decades, survival has continued to improve in both non-metastatic^3^ and metastatic breast cancers (MBC),^4–6^ which nonetheless remain incurable.

Treatment recommendations ought to be made on an individual basis and consider hormone receptor (HR) and human epidermal growth factor receptor 2 (HER2) status. Indeed, the luminal subtype (HR+/HER2−) represents about 70% of all breast cancers. Some studies have shown that, even though no difference was found between chemotherapy (CT) or endocrine therapy (ET) in terms of survival, ET is a better choice since it is less toxic.^7,8^ Some clinical trials showed good progression-free survival (PFS) for ET alone and an increase in PFS for ET in association with targeted therapies.^9–11^ According to international guidelines, ET should be the preferred option in luminal MBC, even in the presence of visceral disease, unless there is a visceral crisis or proof of endocrine resistance.^12–14^

Despite these data, some recent studies have shown that guidelines are not always followed in daily practice and that medical practices vary considerably.^15–19^ However, to date, none of these studies has been done using population-based data.

In this population-based study, which takes into account all of the women living in Cote d’Or (Côte d’Or Breast and Gynaecological Cancer Registry), we investigated the effect of the first-line treatment (ET or CT) on PFS and overall survival (OS) in luminal MBC patients.

## Methods

### Study population and data collection

A population-based study was undertaken using data from the Côte d’Or Breast and Gynaecological Cancer Registry. This registry records all cases of breast and gynaecological cancers in women living in the department of Côte d’Or at the time of diagnosis of the primary cancer, and it is the only registry in France focusing on these cancers. Women diagnosed with HR+/HER2−, MBC from January 1998 to December 2016 were retrospectively selected in the registry data and included in this study. Only invasive ductal or lobular carcinomas were included. HR and HER2 status were determined using immunohistochemistry, completed by a fluorescent in situ hybridisation exam in patients with an equivocal result. Pathologists in Côte d’Or usually classify a patient as RH+ when one of the 2 receptors (oestrogen receptor or progesterone receptor) is >10%. HER2 status was retrospectively determined for cases diagnosed before routine analyses. Women who had metastasis after 2016, those who did not receive a systemic anti-metastatic treatment and those who had concurrent CT and ET in the first line were excluded.

Comorbidities were defined according to the Charlson Comorbidity Index (CCI)^20^ and were classified into two groups: no comorbidities (CCI = 0) and at least one comorbidity (CCI > 0). In accordance with European guidelines, we defined primary endocrine resistance as: relapse while on the first 2 years of adjuvant ET. Secondary endocrine resistance was defined as: relapse while on adjuvant ET but after the first 2 years or relapse within 12 months of completing adjuvant ET.^12^ Furthermore, we described the characteristics of the initial systemic therapies.

Only anonymised data were used for this study. The registry has the necessary regulatory agreements from the French Data Protection Authority (Commission nationale de l’informatique et des libertés (CNIL)) to use patients’ data. The CNIL ensures that data privacy laws are respected (CNIL authorisation number DR-2012-038).

### Primary and secondary outcomes

The primary outcome was PFS defined as the time from initiation of the first systemic therapy for metastatic disease to disease progression or death, whichever occurred first. Progression, as determined by the DATECAN initiative,^21^ was defined as progression of the initial metastases or the occurrence of new metastases.

Our secondary outcome was OS, which was defined as the time from initiation of the first systemic anti-metastatic therapy (CT or ET) to death.

Women alive without progression were censored at the end of the study period (December 31, 2017).

### Statistical analyses

We used chi-square tests or Fisher’s exact tests for categorical variables and Wilcoxon rank-sum test for continuous variables to compare baseline characteristics (patient characteristics, primary tumour characteristics, adjuvant therapies (radiotherapy, CT and ET before the diagnosis of metastasis) and metastasis characteristics) between patients with initial CT and those with initial ET.

Survival outcomes were estimated using the Kaplan–Meier method and compared between groups using the log-rank statistic.

Differences in PFS and OS between patients initially treated with CT or ET were analysed using Cox proportional hazards models after adjustment for factors with a *P* value < 0.2 in univariate analyses. We did not take adjuvant treatments into account in the multivariate analyses because these variables only concern secondary metastases. Moreover, because we only studied metastatic cancers, we did not take the tumour–node stage of the initial tumour into account in the multivariate analyses.

The proportional hazards assumption was checked for each variable using Schoenfeld Residuals tests, and for each factor that did not meet the assumption, we included an interaction with time in the model. We also tested interactions and correlations (*ρ* < 0.7) between the different variables.

To determine the association between prognostic factors and survival outcomes, we fitted univariate Cox proportional hazards models. Prognostic factors were age at diagnosis of MBC (<65 years/≥65 years), CCI (CCI = 0/CCI > 0), menopausal status (premenopausal/postmenopausal), primary tumour type (invasive ductal/lobular carcinomas), primary metastatic disease (yes/no), initial metastatic sites (bone only, visceral or multiple), primary endocrine resistance (yes/no) and year of primary tumour diagnosis (<2008/≥2008). We chose 2008 as a cut-off because changes in practices and in the definition of metastatic disease by the registry occurred in 2008. More specifically, HR/HER2 status became a more important factor in therapeutic decision-making, and before 2008, cancer stage was determined in the registry using the fifth edition of the American Joint Committee for Cancer (AJCC) tumour–node–metastasis (TNM) classification of malignant tumours while the sixth edition was used after 2008.

In the first sensitivity analysis, we used a propensity score (PS) to consider the subjectivity of the physician’s choice^22^ and to adjust on the variables that were different between the two groups of patients (those who underwent initial ET and those who underwent initial CT). Each variable that differed between patients with initial CT and initial ET was included in the multivariate logistic model. First, each patient treated with initial CT was matched with a patient who underwent initial ET with the same characteristics using the PS. Nearest-neighbour matching was used, with a maximal PS distance between matched subjects (calliper) of 0.1; unpaired patients were excluded.

We then performed a second sensitivity analysis in which patients with initial CT who were given maintenance ET before progression were censored at the start of maintenance ET. Similar to a previous study,^18^ this analysis was done to compare the group of patients who were exclusively treated by ET and the others exclusively treated by CT, in an attempt to override the effect of maintenance ET.

We then performed a third sensitivity analysis in which ET was considered a time-dependent variable (taking into account ET no matter when ET was administered, without censoring on maintenance ET). In the initial ET group, ET was started the first day of the follow-up, and in the initial CT group, ET was started when the maintenance ET started.

Statistical analysis was performed using the SAS 9.4 software (SAS Institute, Cary, NC, USA). Statistical significance was set at a *P* value of <0.05.

## Results

### Patient characteristics

**Fig. 1:**
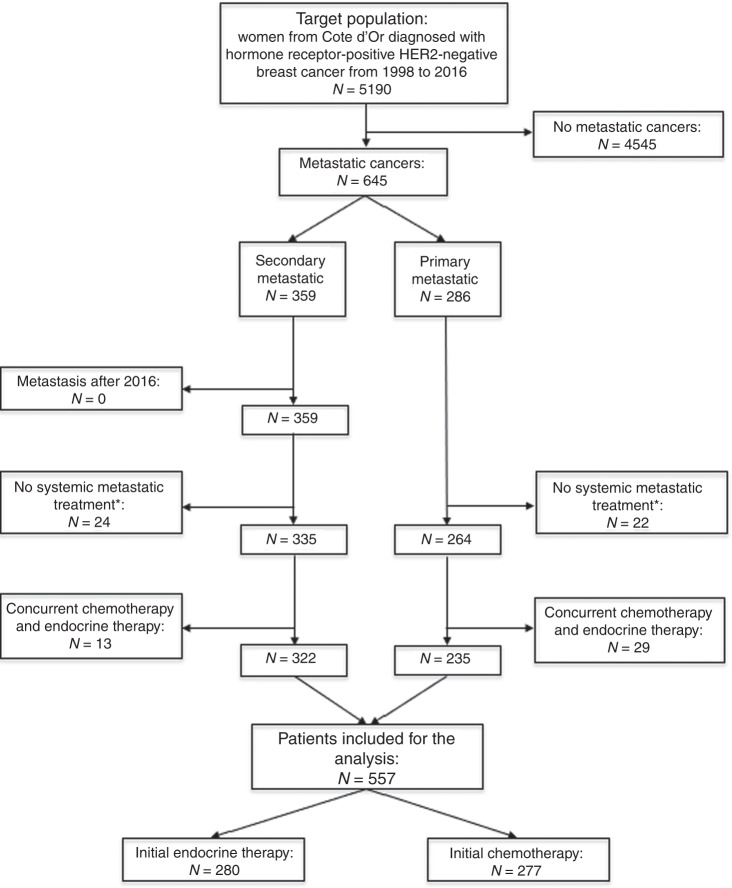
Flowchart of the study population. Asterisk (*): patients who received exclusive comfort treatment or those who received only surgery and/or radiotherapy as treatment for metastatic cancer.

Altogether, 5190 women from Côte d’Or were diagnosed with HR-positive, HER2-negative breast cancer from 1998 to 2016, and 645 cases were or became MBC. Among these, 557 patients received a systemic anti-metastatic treatment and were analysed (277 first-line CT and 280 first-line ET) (Fig. 1).

These patients had a median age of 65 years [interquartile range (IQR): 55–77 years] when MBC was diagnosed. The median follow-up was 25 months (IQR: 13–45 months). The median PFS was 13 months (95% confidence interval (CI) 12–14) and the median OS was 26 months (95% CI 23–29). At the end of the study period (December 31, 2017), 95% of all patients (531/557) had progressed and 83% (463/557) had died.

Altogether, 50% of patients were treated with initial CT and 50% of patients received initial ET; their characteristics are summarised in Table 1. There were no statistical differences between the two groups concerning primary tumour characteristics (diagnostic year, histological subtype, HR status and node status). Patients treated with initial CT were younger, had fewer comorbidities and were given more adjuvant therapies than those treated with initial ET. Initial ET was more frequently used in women with primary MBC and bone metastases only; there was less endocrine resistance in this group.

**Table 1:** Patient demographics and disease characteristics of the endocrine and chemotherapy groups.

Characteristics	Initial chemotherapy, *N* = 277, *n* (%)	Initial endocrine therapy, *N* = 280, *n* (%)	*P*
Age at MBC diagnosis			
Median in years (IQR)	61 (50–69)	73 (61–80)	<**0.0001**^a^
<65 years	170 (61)	97 (35)	<**0.0001**
≥65 years	107 (39)	183 (65)	
Menopausal status			**<0.0001**
Premenopausal	83 (30)	35 (12)	
Postmenopausal	194 (70)	245 (88)	
Charlson Comorbidity Index (CCI)			
CCI = 0	178 (64)	130 (46)	<**0.0001**
CCI > 0	99 (36)	150 (54)	
Primary tumour diagnostic year			
<2008	97 (35)	119 (42)	0.07
≥2008	180 (65)	161 (58)	
Primary tumour type			0.2543
Ductal	231 (83)	223 (80)	
Lobular	46 (17)	57 (20)	
Hormone receptor status			
ER positive	269 (97)	276 (99)	0.2355
PR positive	199 (72)	208 (74)	0.2388
Primary tumour stage^b^			0.1053
T1–T2	187 (67)	172 (61)	
T3–T4	88 (32)	108 (39)	
Unknown	2 (1)	0	
Node status^b^			0.9667
Node negative	139 (50)	140 (50)	
Node positive	132 (48)	132 (48)	
Unknown	6 (2)	8 (2)	
Adjuvant systemic therapy^c^			
Endocrine therapy	164 (91)	105 (74)	**<0.0001**
Chemotherapy	141 (78)	82 (58)	<**0.0001**
None	6 (3)	24 (17)	<**0.0001**^d^
Adjuvant radiotherapy^c^			0.3244
Yes	163 (90)	122 (87)	
No	18 (10)	19 (13)	
Primary metastatic disease			
Yes	96 (35)	139 (50)	**0.0002**
No	181 (65)	141 (50)	
Metastasis-free interval^c^			0.1423
<24 months	26 (14)	29 (21)	
≥24 months	155 (86)	112 (79)	
Initial metastatic sites			**<0.0001**
Bone only	62 (22)	136 (49)	
Visceral	90 (33)	66 (23)	
Multiple	125 (45)	78 (28)	
Endocrine resistance			**0.0003**
Primary	50 (18)	35 (13)	
Secondary	74 (27)	45 (16)	
Hormone responsive	153 (55)	200 (71)	

Bold values indicate statistically significant results.

*n* number, *IQR* interquartile range, *MBC* metastatic breast cancer, *ER* oestrogen receptor, *PR* progesterone receptor.

^a^Wilcoxon rank-sum test.

^b^Fifth edition before 2008 and sixth edition since 2008 of the TNM classification of malignant tumours.

^c^Only on secondary metastatic diseases.

^d^Fisher’s exact test.

### Treatments

Among patients with initial ET, 72% (203/280) were treated with aromatase inhibitors, 24% (66/280) with anti-oestrogens (tamoxifen, fulvestrant) and 4% (11/280) with chemical or surgical castration.

Among patients with initial CT, 32% (88/277) were given anthracycline-based treatments and 60% (165/277) were given taxane-based treatments. Altogether, 53% (146/277) changed to maintenance ET, and among these 60% (88/146) changed within 6 months of starting CT.

### Analysis of survival outcomes

**Fig. 2:**
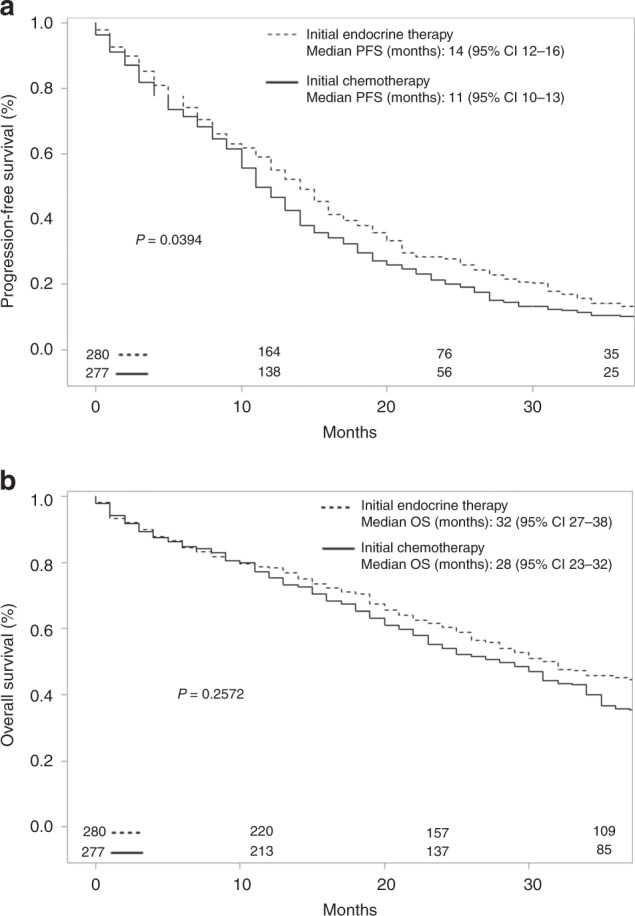
a) Progression-free survival and b) overall survival according to initial systemic anti-metastatic therapy of patients with hormone receptor-positive metastatic breast cancer. CI confidence interval, PFS progression-free survival, OS overall.

PFS and OS were compared in patients who received ET or CT as the initial systemic anti-metastatic therapy. The median PFS of patients treated with initial ET was 14 months (95% CI 12–16) and the median PFS of those treated with initial CT was 11 months (95% CI 10–13; *P* = 0.0394) (Fig. 2a). The median OS of patients treated with initial ET was 32 months (95% CI 27–38) and the median OS of those treated with initial CT was 28 months (95% CI 23–32; *P* = 0.2572) (Fig. 2b).

Adjustment factors for the multivariate model for PFS were primary metastatic disease, initial metastatic sites, menopausal status, CCI and primary endocrine resistance. PFS was significantly better in patients treated with initial ET than in those with initial CT, with a hazard ratio (HR) of 0.83 (95% CI 0.69–0.99). Adjustment factors for the multivariate model for OS were: age at MBC diagnosis, menopausal status, CCI, primary metastatic disease, initial metastatic sites, and primary endocrine resistance. Because “primary metastatic disease” did not meet the proportional hazards assumption, we included an interaction between “primary metastatic disease” and time in the multivariate model. OS was significantly better in patients treated with initial ET than in those with initial CT, with an HR of 0.71 (95% CI 0.58–0.86) (Table 2).

**Table 2:** Univariate and multivariate analysis of survival outcomes of hormone receptor-positive metastatic breast cancer patients.

Characteristics	PFS				OS			
	Univariate HR (95% CI)	*P*	Multivariate HR (95% CI)	*P*	Univariate HR (95% CI)	*P*	Multivariate^a^ HR (95% CI)	*P*
Initial systemic therapy								
Initial chemotherapy	1	**0.0452**	1	**0.0472**	1	0.2623	1	**0.0004**
Initial endocrine therapy	0.84 (0.70–0.99)		0.83 (0.69–0.99)		0.90 (0.75–1.08)		0.71 (0.58–0.86)	
Primary metastatic disease								
No	1	<**0.0001**	1	**0.0004**	1	0.0765	—	—
Yes	0.69 (0.58–0.83)		0.71 (0.59–0.86)		0.85 (0.70–1.02)		—	
Initial metastatic sites								
Bone only	1	**0.0016**	1	**0.0158**	1	**0.0007**	1	0.1673
Visceral or multiple	1.34 (1.12–1.60)		1.26 (1.04–1.51)		1.40 (1.15–1.69)		1.15 (0.94–1.41)	
Hormonal status								
Premenopausal	1	0.1695	1	0.2323	1	**0.0001**	1	0.1434
Postmenopausal	1.16 (0.94–1.43)		1.15 (0.92–1.43)		1.55 (1.29–1.87)		1.23 (0.93–1.63)	
Charlson Comorbidity Index (CCI)								
CCI = 0	1	**0.0368**	1	**0.0297**	1	<**0.0001**	1	**0.0195**
CCI > 0	1.20 (1.01–1.42)		1.22 (1.02–1.47)		1.51 (1.23–1.84)		1.27 (1.04–1.55)	
Primary endocrine resistance								
No	1	**0.0215**	1	0.4450	1	0.0565	1	0.2596
Yes	1.32 (1.04–1.67)		1.10 (0.86–1.42)		1.27 (0.99–1.63)		1.17 (0.89–1.53)	
Age at MBC diagnosis								
<65 years	1	0.5689	—	—	1	**<0.0001**	1	**0.0147**
≥65 years	1.05 (0.89–1.25)		—		1.64 (1.36–1.98)		1.32 (1.06–1.65)	
Primary tumour type								
Ductal	1	0.7039	—	—	1	0.8611	—	—
Lobular	1.04 (0.84–1.30)		—		1.02 (0.81-1.29)		—	
Primary tumour diagnosis year								
<2008	1	0.2060	—	—	1	0.8779	—	—
≥2008	1.12 (0.94–1.34)		—		1.01 (0.84–1.22)		—	

Bold values indicate statistically significant results.

*MBC* metastatic breast cancer.

^a^With an interaction between “primary metastatic disease” and time.

In the analyses with PS (first sensitivity analysis), matched cases (*N* = 296) showed an HR = 0.87 (95% CI 0.70–1.07) for PFS and an HR of 0.79 (95% CI 0.63–0.99) for OS.

In the second sensitivity analysis, in which patients of the CT group with maintenance ET were censored, PFS and OS were significantly better in patients treated with initial ET than in those with initial CT with an HR of 0.72 (95% CI 0.56–0.92) and 0.61 (95% CI 0.51–0.74), respectively.

In the third sensitivity analysis, in which ET was considered a time-dependent variable, PFS was not significantly better in patients treated with initial ET than in those with initial CT, with an HR of 0.84 (95% CI 0.70–1.01), and OS was significantly better in patients treated with initial ET than in those with initial CT, with an HR of 0.69 (95% CI 0.57–0.84).

## Discussion

The results of this population-based study showed that PFS and OS in patients initially treated with ET were improved significantly when compared to patients treated with initial CT as the systemic anti-metastatic treatment (HR = 0.83; 95% CI 0.69–0.99 for PFS and HR = 0.71; 95% CI 0.58–0.86 for OS). It is worth noting that the PFS in our initial ET group was comparable to that of patients with initial ET in other large clinical trials.^9,23^

In order to limit the indication bias that may have influenced the first sensitivity analysis, we created PSs. With the matching method, the only difference between the two smaller groups was the initial treatment. This created a framework similar to a randomised study but only for the variables selected to create the PS. The results of this analysis reinforced our findings regarding survival outcomes considering that the matched groups were comparable (Table 3) and the HRs were similar, even if the power was lower (HR = 0.87; 95% CI 0.70–1.07 for PFS, and HR = 0.79; 95% CI 0.63–0.99 for OS).

**Table 3:** Distribution of characteristics of patients matched with the propensity score according to the initial systemic anti-metastatic therapy.

Characteristics	Initial chemotherapy, *N* = 186, *n* (%)	Initial endocrine therapy, *N* = 186, *n* (%)	*P*
Age at diagnostic MBC			0.9170
<65 years	84 (45)	83 (45)	
≥65 years	102 (55)	103 (55)	
Hormonal status			0.7861
Premenopausal	34 (18)	32 (18)	
Postmenopausal	152 (82)	154 (82)	
Charlson Comorbidity Index (CCI)			0.7524
CCI = 0	110 (58)	107 (56)	
CCI > 0	76 (42)	79 (44)	
Primary tumour type			0.7950
Ductal	150 (81)	148 (80)	
Lobular	36 (19)	38 (20)	
Primary metastatic disease			0.5678
Yes	110 (59)	105 (56)	
No	76 (41)	81 (44)	
Initial metastatic sites			0.8044
Bone only	60 (32)	62 (33)	
Visceral	53 (29)	57 (31)	
Multiple	73 (39)	67 (36)	
Endocrine resistance			0.1161
Primary	108 (58)	125 (67)	
Secondary	28 (15)	27 (15)	
Hormone responsive	50 (27)	34 (18)	

*n* number, *MBC* metastatic breast cancer.

There are two recent real-life studies on this subject, one by Lobbezzo et al. in the Netherlands^18^ and another by Jacquet et al. in France.^19^ Like in Lobbezzo et al.’s study, in which patients in the CT group who underwent maintenance ET were censored to avoid the effect of maintenance ET, we thus performed a second sensitivity analysis which also showed that PFS and OS were significantly better in patients initially treated with ET (respectively, HR = 0.72; 95% CI 0.56–0.92 and HR = 0.61; 95% CI 0.51–0.74). However, our results also supported our assumption that this analysis contained a potential bias because censoring on maintenance ET was informative: in calculating the survival time of CT patients, it was reduced to those with maintenance ET, thus favouring the ET group when comparing survival time. As a consequence, censorship could have deteriorated PFS in the CT group. This bias may have been stronger in our study because 53% of our CT patients received maintenance ET compared with only 8% in Lobbezzo et al.^18^ On the other hand, median OS and median PFS in the initial CT group was much lower in Lobbezzo et al.’s study than in our study. This could be explained by differences in baseline population characteristics. Moreover, Lobbezzo et al. included patients prior to introduction of daily bevacizumab, whereas our study included patients subsequently; the use of bevacizumab could have increased PFS in the initial CT group.^24^ To avoid the potential bias due to informative censoring, we also performed a third sensitivity analysis in which ET was considered a time-dependent variable, without censoring on maintenance ET. This analysis gave us an estimate of the effect of ET no matter when ET was administered and showed results similar to the primary analysis (HR = 0.84, 95% CI 0.70–1.01for PFS and HR = 0.69, 95% CI 0.57–0.84 for OS). However, in this analysis, the introduction of a new variable (time-dependent variable) reduced the power, which may explain why this result was not statistically significant for PFS. Of course, this analysis is not perfectly comparable to the primary analysis as we have not only considered initial ET but also maintenance ET.

Contrary to our study, Jacquet et al. found no significant differences in PFS and OS between initial ET and initial CT. However, the two populations are not comparable; indeed they included only diseases sensitive to aromatase inhibitors, whereas we included all patients, even the hormone-resistant ones.

Our results also evidenced that the treatments administered to patients in Côte d’Or were considerably different from the treatments recommended by international guidelines.^12–14^ Half of our study population received initial CT even though only 15% of these patients had clinical primary endocrine resistance. The population-based nature of our study could explain the large proportion of initial CT in comparison with hospital cohorts given that we recruited patients from different types of medical establishments. Furthermore, the first international consensus guidelines for advanced breast cancer were published relatively recently, in 2012.^25^ In our study, the proportion of patients receiving initial CT continued to increase after 2008 despite the publication of new guidelines.

Almost two-thirds of patients who changed to maintenance ET did so within 6 months of starting CT. We could suppose that this was partly related to toxicity but were not able to obtain specific information about toxicity or visceral crises.

Our data were obtained from the Côte d’Or Breast and Gynaecological Cancer Registry, which respects a strict data quality control policy including regular checks to ensure complete follow-up for every patient. Our high-quality data is not subject to the selection bias that can be observed with hospital cohorts. However, the registry covers a relatively small geographic area, obliging us to study the data over a long period in order to obtain a large enough sample. Diagnostic procedures and therapies have improved since the start of the study. In addition, the tumour classification changed: we used the fifth edition of the AJCC TNM classification of malignant tumours before 2008, and the sixth edition after 2008. Consequently, before 2008 we considered a few metastatic tumours that would have been considered N3 after 2008. Furthermore, the level of intensity of HRs was poorly documented in the registry in the early years of the study because clinicians did not take this information into account when choosing the first systemic anti-metastatic treatment. Likewise, we were unable to include the performance status because this information was not routinely collected in our register. However, we did take into account the CCI, which provides a good indicator of general health.

Our main results, which show a statistically significant difference in favour of ET, may have several explanations. First, the patients included in our study are all HR+ and who were therefore more sensitive to ET. In addition, HR+/HER2− MBC are slower replicating cancers, which are therefore less sensitive to cytotoxic treatment than quicker replicating cancers.

Moreover, the results of the first studies on new targeted therapies (cyclin-dependent kinase 4/6 inhibitors), which are expensive but less toxic, showed an ability to prolong PFS.^9,23^ However, the factors predicting response have not yet been identified. In our study, very few patients received targeted therapies because they are fairly recent relative to our follow-up period. Further studies will be needed to determine the best strategy for treating patients with HR+/HER2− MBC.

## Conclusion

In conclusion, in this population-based study, we have performed a primary analysis along with three sensitivity analyses (PS, censoring at the start of the maintenance ET and considering ET as a time-dependent variable) to overcome potential biases. All these analyses showed very similar results indicating that initial treatment with ET improves survival in patients with HR-positive HER2-negative MBC, as compared with initial treatment with CT. Knowing that ET causes fewer side effects, we suggest that international guidelines should be followed more closely, and more extensively disseminated, with a clarification of the definition of visceral crisis.

## Data Availability

The data used for this study were extracted from the Côte d’Or Breast and Gynaecological Cancer Registry database.
